# Estimation of Atherosclerotic Cardiovascular Disease Risk Among Patients in the Veterans Affairs Health Care System

**DOI:** 10.1001/jamanetworkopen.2020.8236

**Published:** 2020-07-14

**Authors:** Jason L. Vassy, Bing Lu, Yuk-Lam Ho, Ashley Galloway, Sridharan Raghavan, Jacqueline Honerlaw, Laura Tarko, John Russo, Saadia Qazi, Ariela R. Orkaby, Vidisha Tanukonda, Luc Djousse, J. Michael Gaziano, David R. Gagnon, Kelly Cho, Peter W. F. Wilson

**Affiliations:** 1 Veterans Affairs Boston Healthcare System, Boston, Massachusetts; 2 Department of Medicine, Brigham and Women’s Hospital, Harvard Medical School, Boston, Massachusetts; 3 Veterans Affairs Eastern Colorado Healthcare System, Aurora; 4 Division of Hospital Medicine, University of Colorado School of Medicine, Aurora; 5 Colorado Cardiovascular Outcomes Research Consortium, Aurora; 6 Landmark College, Putney, Vermont; 7 Department of Biostatistics, Boston University School of Public Health, Boston, Massachusetts; 8 Atlanta Veterans Affairs Medical Center, Decatur, Georgia; 9 Division of Cardiology, Emory University School of Medicine, Atlanta, Georgia; 10 Rollins School of Public Health, Department of Epidemiology, Emory University, Atlanta, Georgia

## Abstract

**Importance:**

Current guidelines recommend statin therapy for millions of US residents for the primary prevention of atherosclerotic cardiovascular disease (ASCVD). It is unclear whether traditional prediction models that do not account for current widespread statin use are sufficient for risk assessment.

**Objectives:**

To examine the performance of the Pooled Cohort Equations (PCE) for 5-year ASCVD risk estimation in a contemporary cohort and to test the hypothesis that inclusion of statin therapy improves model performance.

**Design, Setting, and Participants:**

This cohort study included adult patients in the Veterans Affairs health care system without baseline ASCVD. Using national electronic health record data, 3 Cox proportional hazards models were developed to estimate 5-year ASCVD risk, as follows: the variables and published β coefficients from the PCE (model 1), the PCE variables with cohort-derived β coefficients (model 2), and model 2 plus baseline statin use (model 3). Data were collected from January 2002 to December 2012 and analyzed from June 2016 to March 2020.

**Exposures:**

Traditional ASCVD risk factors from the PCE plus baseline statin use.

**Main Outcomes and Measures:**

Incident ASCVD and ASCVD mortality.

**Results:**

Of 1 672 336 patients in the cohort (mean [SD] baseline age 58.0 [13.8] years, 1 575 163 [94.2%] men, 1 383 993 [82.8%] white), 312 155 (18.7%) were receiving statin therapy at baseline. During 5 years of follow-up, 66 605 (4.0%) experienced an ASCVD event, and 31 878 (1.9%) experienced ASCVD death. Compared with the original PCE, the cohort-derived model did not improve model discrimination in any of the 4 age-sex strata but did improve model calibration. The PCE overestimated ASCVD risk compared with the cohort-derived model; 211 237 of 1 136 161 white men (18.6%), 29 634 of 218 463 black men (13.6%), 1741 of 44 399 white women (3.9%), and 836 of 16 034 black women (5.2%) would be potentially eligible for statin therapy under the PCE but not the cohort-derived model. When added to the cohort-derived model, baseline statin therapy was associated with a 7% (95% CI, 5%-9%) lower relative risk of ASCVD and a 25% (95% CI, 23%-28%) lower relative risk for ASCVD death.

**Conclusions and Relevance:**

In this study, lower than expected rates of incident ASCVD events in a contemporary national cohort were observed. The PCE overestimated ASCVD risk, and more than 15% of patients would be potentially eligible for statin therapy based on the PCE but not on a cohort-derived model. In the statin era, health care professionals and systems should base ASCVD risk assessment on models calibrated to their patient populations.

## Key Points

**Question:**

Are current risk prediction models accurate at estimating risk of initial atherosclerotic cardiovascular (ASCVD) events in veterans?

**Findings:**

In this cohort study of 1 672 336 veterans with an outpatient visit between 2002 and 2007, the 2013 American College of Cardiology/American Heart Association model overestimated absolute risk of ASCVD during 5 years of follow-up. Statin use was associated with 7% lower relative risk of ASCVD and 25% lower relative risk of ASCVD mortality.

**Meaning:**

The findings of this study suggest that reestimation and the inclusion of statin use in ASCVD prediction models might be needed for their appropriate use in a health care system.

## Teaser

This cohort study examines the performance of the Pooled Cohort Equations (PCE) for 5-year atherosclerotic cardiovascular disease risk estimation in a contemporary cohort and tests the hypothesis that inclusion of statin therapy improves model performance.

## Introduction

Atherosclerotic cardiovascular disease (ASCVD) continues to be the leading cause of death and disability in the United States, and predicting and reducing ASCVD risk remains a paramount priority for clinical medicine and public health. In 2001, a National Heart, Lung, and Blood Institute workshop concluded that Framingham Risk Score variables—age, male sex, smoking, systolic blood pressure, antihypertensive therapy, and total and high-density lipoprotein (HDL) cholesterol—were key risk factors that could guide clinical decision-making about lipid-lowering therapy for primary ASCVD prevention.^1^ In 2013, the Pooled Cohort Equations (PCE) used data from more diverse US populations and extended ASCVD risk algorithms to men and women of white and black race.^2^ Subsequentlyy, the 2013 American College of Cardiology/American Heart Association (ACC/AHA) guidelines recommended using PCE risk categories to guide the intensity of statin therapy.^3^ The 2018 update to these guidelines continued to recommend the use of the PCE in risk stratification but added other risk enhancing factors, such as chronic kidney or inflammatory diseases, to consider when making decisions about statin therapy for ASCVD prevention.^4^

More than 140 million US residents are now prescribed statin therapy,^5^ and almost half of US adults are potentially eligible to receive statin therapy for ASCVD prevention according to ACC/AHA guidelines.^2,6^ As the use of statins in the contemporary practice of medicine has become more widespread, it is unclear whether traditional prediction models that do not account for statin use are sufficiently informative for risk assessment. A prior report of national data from the Veterans Health Administration (VA)^7^ found that the PCE significantly overestimated ASCVD risk, but it did not derive new effect estimates for PCE terms or evaluate the inclusion of statin therapy in risk estimation. Here, we used national VA electronic health record data to examine the performance of the PCE for 5-year ASCVD risk estimation in a contemporary patient cohort and tested the hypothesis that the addition of statin therapy improves model performance.

## Methods

### Research Ethics Statement

This study was reviewed and approved by the institutional review boards of Emory University and VA Boston Healthcare System. This study was restricted to secondary data analysis, and thus, the requirement for informed consent from participants was waived. This manuscript follows the Strengthening the Reporting of Observational Studies in Epidemiology (STROBE) reporting guideline for cohort studies.

### Data Sources

The national VA Corporate Data Warehouse^8^ was used to create a virtual study cohort of veterans who are regular users of the VA health care system. Data from the VA Corporate Data Warehouse were linked to data from the Centers for Medicaid & Medicare Services and National Death Index databases using scrambled social security numbers.^9,10^

### Population

We created a prospective cohort study of incident ASCVD events from national VA data, using previously described methods.^11^ In brief, this cohort included all VA patients aged at least 18 years with at least 1 primary care visit at a VA facility who had at least 1 outpatient lipid result between 2002 and 2007 and a blood pressure measurement within 30 days of this index lipid testing, a criterion based on our prior work demonstrating that such a restriction limits potential bias within this cohort.^11^ The date of index lipid determination served as the baseline date of entry to the cohort.

Individuals were excluded from the cohort if they had a history of HIV infection, cancer, significant kidney or liver disease, schizophrenia, or dementia at baseline (eTable 1 in the Supplement). Given the focus on the performance of the 2013 ACC/AHA PCE, which consists of 4 separate risk models for white and black men and women, we excluded any individual with a different race or ethnicity. In the present analysis of primary ASCVD events, individuals were also excluded if they had baseline history of cardiovascular disease, defined as the presence of a code for any of the following: (1) stroke or cerebrovascular disease, including hemorrhagic stroke, ischemic stroke, and transient ischemic attack (*International Classification of Diseases, Ninth Revision *[*ICD*-*9*] codes 430-438 or meeting the ischemic stroke algorithm described below); (2) coronary heart disease or coronary artery disease, including myocardial infarction (MI), angina, and coronary insufficiency (*ICD-9* codes 410-414 or 429.2 or meeting the MI algorithm described below); (3) peripheral vascular disease (*ICD*-*9* codes 440.20-440.4 or 443.9 or history of amputation [eTable 1 in the Supplement]); and (4) congestive heart failure (*ICD*-*9* code 428).

The eFigure in the Supplement illustrates the cohort creation. Of 8 401 688 patients who accessed VA care from 2002 to 2007, 1 672 336 (19.9%) met the cohort inclusion criteria. The most common reasons for exclusion were absence of a lipid result from 2000 to 2007 (3 713 614 patients [44.2%]) and significant comorbidities, including prevalent CVD (2 349 064 patients [28.0%]).

### Outcomes

The primary outcomes included time to incident ASCVD event and ASCVD mortality. Incident ASCVD events were defined as a composite outcome of ischemic stroke, MI, and coronary death. To identify stroke and MI, we adapted a phenotyping algorithm designed to identify prevalent cases, using a combination of *ICD* codes from both VA and Center for Medicare & Medicaid Services data sources, natural language processing, and medical record review labels.^12^ These phenotyping methods resulted in probability of ischemic stroke and MI for each participant. A predicted probability of ischemic stroke of at least 0.770 and of MI of least 0.765 was defined as a definite case to ensure positive predictive values of at least 90% compared with expert clinician medical record review. Vital status and date of death, if applicable, were derived from the National Death Index. We defined ASCVD mortality as having any of the following *ICD*-*10*-*CM* diagnosis codes as the National Death Index primary cause of death: I10, I11, I13, I16, I20 to I25, I46, I63, I67, I70, I74, I75, and G45.

### Risk Factors for ASCVD

Guided by the PCE, we considered the following traditional risk factors: age, sex, race, total cholesterol, HDL cholesterol, diabetes, systolic blood pressure, blood pressure treatment, and smoking history, all assessed at the index date. Demographic variables, such as age (difference between the index date and date of birth), race (categorized as black or white), and sex, were derived from administrative data sources within the Corporate Data Warehouse. Diabetes was defined as the use of a diabetes medication before the baseline date (eTable 1 in the Supplement) plus either 2 *ICD*-*9*-*CM* diagnosis codes 250.xx or the use of 1 or more 250.xx codes in combination with a VA primary care visit.^13^ Blood pressure treatment was defined as an active prescription on the baseline date for at least 1 of the following medication classes, taken as a single agent or in combination formulations: diuretics, angiotensin-converting enzyme inhibitors, angiotensin II receptor inhibitors, α-blockers, β-blockers, and calcium channel blockers. Consistent with the PCE, smoking history was categorized as current or not current (a composite of never and former smoking) with an algorithm developed and validated within the VA medical record data.^14^ We also assessed the role of baseline statin therapy, defined by an active statin prescription at the time of baseline lipid measurement (eTable 1 in the Supplement).

### Statistical Analysis

**Figure 1.**
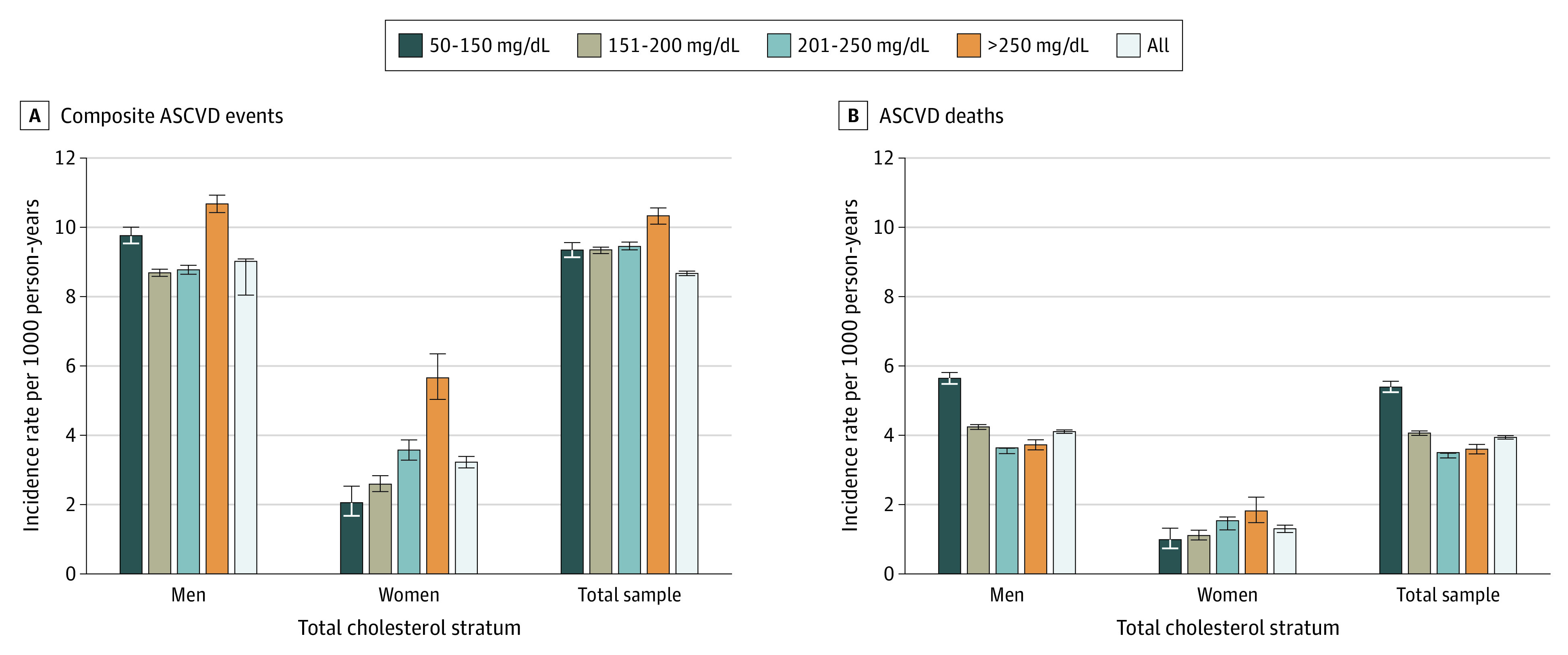
Incidence Rates per 1000 Person-Years for Composite Outcome and Atherosclerotic Cardiovascular Disease (ASCVD) Death Whiskers indicate 95% CIs. To convert total cholesterol to millimoles per liter, multiply by 0.0259.

Analysis was performed from June 2016 to March 2020. Baseline descriptive statistics of the study sample, such as the minimum, maximum, median, and mean for each continuous variable and frequency table for each categorical variable, were initially analyzed to summarize the data and detect outliers and missing values. Missing data were uncommon, and individuals with missing data for risk factors were excluded from analysis. We then fitted Cox proportional hazard models to estimate the effect of each PCE risk factor on 5-year and 10-year ASCVD events and ASCVD mortality, with and without baseline statin treatment. Individuals without the primary outcome (composite ASCVD event or ASCVD mortality) were censored 5 or 10 years after baseline in the corresponding analysis. We used a 5-year estimation timeline as our primary analysis because of the large number of ASCVD events observed during that interval and to limit potential misclassification of statin and blood pressure medication treatment status for the participants after the baseline date. We present 10-year models as supplementary analyses. In all models, we categorized total cholesterol in 5 separate clinically relevant strata (50-150 mg/dL, 151-200 mg/dL, 201-250 mg/dL, and >250 mg/dL [to convert to millimoles per liter, multiply by 0.0259]) because of its U-shaped association with mortality observed in this population (Figure 1).

To evaluate the performance of the PCE in this cohort, we developed separate sex- and race-specific Cox proportional hazards models estimating the 5-year risk of ASCVD and ASCVD mortality. We developed 3 successive models, as follows: (1) the published 2013 ACC/AHA variables (including log transformations and interactions terms) and β coefficients from the 2013 ACC/AHA prediction equations (model 1)^2^; (2) these variables fit to cohort-specific Cox regression models for white men, black men, white women, and black women separately (model 2); and (3) model 2 plus baseline statin use (model 3). Harrell C statistics and their SDs were calculated to evaluate the ability of the models to separate those who experienced events from those who did not (ie, discrimination).^15^ To visualize model calibration, we plotted observed 5-year Kaplan-Meier rates according to the deciles of model estimated rates, using previously reported 5-year survival estimates from the original development of the PCE.^2,16^ Finally, we examined the distribution of patients aged 40 to 79 years in clinically meaningful 5-year ASCVD risk categories (ie, <2.5%, 2.5%-3.74%, 3.75%-10%, >10%) according to the original PCE (model 1) and the cohort-specific model (model 2).

We used SAS enterprise guide version 7.1 and SAS version 9.4 software (SAS Institute) for these analyses. Statistical significance was set at α = .05. All tests of statistical significance were 2-tailed.

## Results

In the total eligible cohort of 1 672 336 patients, the mean (SD) baseline age was 58.0 (13.8) years, 1 575 163 patients (94.2%) were men, and 1 383 993 (82.8%) were white individuals. Table 1 shows the baseline characteristics of this cohort, additionally stratified by incident ASCVD event and death. Overall, 331 925 patients (19.9%) were currently smoking at baseline, and 290 092 (17.4%) had diabetes. At baseline, 312 155 (18.7%) had a prescription for statin therapy. The number of participants with a statin prescription at some time during 5 and 10 years of follow-up increased to 899 464 (53.8%) and 1 048 079 (62.7%), respectively. During a mean (SD) follow-up of 4.6 (1.1) years, 66 605 (4.0%) experienced an ASCVD event; during a mean (SD) follow-up of 4.8 (0.7) years, 31 878 (1.9%) experienced ASCVD death. The characteristics of the 1 663 422 participants included in the supplementary analyses of 10-year events, among whom 121 893 (7.3%) experienced an ASCVD event and 74 230 (4.5%) experienced an ASCVD death, appear in eTable 2 in the Supplement.

**Table 1.** Baseline Characteristics and Incident ASCVD Events Among 1 672 336 Veterans

Characteristic	No. (%)				
	Total cohort (N = 1 672 336)	ASCVD event		ASCVD death	
		Yes (n = 66 605)	No (n = 1 605 731)	Yes (n = 31 878)	No (n = 1 640 458)
Age, mean (SD), y	58.0 (13.8)	64.5 (11.5)	57.7 (13.8)	70.2 (11.8)	57.8 (13.7)
Total cholesterol, mean (SD), mg/dL	197.1 (40.1)	198.8 (42.7)	197.0 (40.0)	192.4 (41.8)	197.2 (40.0)
HDL-C, mean (SD), mg/dL	45.9 (14.0)	44.5 (14.3)	45.9 (14.0)	46.5 (15.6)	45.9 (14.0)
Systolic blood pressure, mean (SD), mm Hg	135.7 (18.3)	141.8 (20.8)	135.5 (18.2)	142.3 (21.4)	135.6 (18.3)
Men	1 575 163 (94.2)	65 125 (97.8)	1 510 038 (94.0)	31 258 (98.1)	1 543 905 (94.1)
White patients	1 383 993 (82.8)	55 704 (83.6)	1 328 289 (82.7)	27 599 (86.6)	1 356 394 (82.7)
Current smoking	331 925 (19.9)	13 951 (21.0)	317 974 (19.8)	4074 (12.8)	327 851 (20.0)
Diabetes	290 092 (17.4)	19 596 (29.4)	270 496 (16.9)	8367 (26.3)	281 725 (17.2)
Blood pressure treatment	683 222 (40.9)	38 527 (57.8)	644 695 (40.2)	19 672 (61.7)	663 550 (40.5)
Statin treatment	312 155 (18.7)	15 231 (22.9)	296 924 (18.5)	6706 (21.0)	305 449 (18.6)

Abbreviations: ASCVD, atherosclerotic cardiovascular disease; HDL-C, high-density lipoprotein cholesterol.

SI conversion factor: To convert total cholesterol and HDL-C to millimoles per liter, multiply by 0.0259.

Figure 1 shows the unadjusted incidence rates of ASCVD events and ASCVD death in the cohort overall and separately for men and women. Incidence of ASCVD events was 9.02 (95% CI, 8.05-9.09) events per 1000 person-years in men, 3.22 (95% CI, 3.06-3.39) events per 1000 person-years in women, and 8.67 (95% CI, 8.61-8.74) per 1000 person-years in the overall cohort. Incidence of ASCVD death was 4.10 (95% CI, 4.06-4.15) events per 1000 person-years in men, 1.29 (95% CI, 1.19-1.40) events per 1000 person-years in women, and 3.93 (95% CI, 3.89-3.98) per 1000 person-years in the overall cohort. As shown in Table 2, risk factors for 5-year ASCVD event included increasing age (without statin: hazard ratio [HR], 1.23; 95% CI, 1.22-1.23; *P* < .001; with statin: HR, 1.23; 95% CI, 1.22-1.23; *P* < .001), male sex (women without statin: HR, 0.63; 95% CI, 0.60-0.66; *P* < .001; women with statin: HR, 0.63; 95% CI, 0.60-0.66; *P* < .001), black race (without statin: HR, 1.20; 95% CI, 1.18-1.23; *P* < .001; with statin: HR, 1.19; 95% CI, 1.17-1.22; *P* < .001), diabetes (without statin: HR, 1.61; 95% CI, 1.58-1.63; *P* < .001; with statin: HR, 1.62; 95% CI, 1.59-1.65; *P* < .001), current smoking (without statin: HR, 1.52; 95% CI, 1.49-1.55; *P* < .001; with statin: HR, 1.52; 95% CI, 1.49-1.55; *P* < .001), and antihypertensive therapy (without statin: HR, 1.35; 95% CI, 1.33-1.39; *P* < .001; with statin: HR, 1.37; 95% CI, 1.35-1.39; *P* < .001). Each 10–mm Hg increment in systolic blood pressure was associated with a 9% (95% CI, 9%-10%) greater relative risk for ASCVD (*P* < .001). Compared with the reference range for total cholesterol level of 50 to 150 mg/dL, a level greater than 250 mg/dL was associated with 59% (95% CI, 54%-65%) greater relative risk for ASCVD event in a model not accounting for statin therapy (*P* < .001). When added to the model, statin therapy was associated with 7% (95% CI, 5%-9%) lower ASCVD relative risk (*P* < .001).

**Table 2.** Five-Year Estimation Models for Composite ASCVD Events and ASCVD Mortality, Without and With Statin Therapy

Characteristic	Composite ASCVD events				ASCVD mortality			
	Without statin		With statin		Without statin		With statin	
	HR (95% CI)	*P* value	HR (95% CI)	*P* value	HR (95% CI)	*P* value	HR (95% CI)	*P* value
Age, per 5-y increase	1.23 (1.22-1.23)	<.001	1.23 (1.22-1.23)	<.001	1.51 (1.50-1.52)	<.001	1.51 (1.50-1.52)	<.001
Women vs men	0.63 (0.60-0.66)	<.001	0.63 (0.60-0.66)	<.001	0.58 (0.54-0.63)	<.001	0.58 (0.54-0.63)	<.001
Black race vs white race	1.20 (1.18-1.23)	<.001	1.19 (1.17-1.22)	<.001	1.20 (1.16-1.24)	<.001	1.17 (1.13-1.21)	<.001
Diabetes vs no diabetes	1.61 (1.58-1.63)	<.001	1.62 (1.59-1.65)	<.001	1.32 (1.28-1.35)	<.001	1.36 (1.33-1.40)	<.001
Current smoking vs no current smoking	1.52 (1.49-1.55)	<.001	1.52 (1.49-1.55)	<.001	1.24 (1.19-1.28)	<.001	1.22 (1.18-1.26)	<.001
Total cholesterol strata, mg/dL								
50-150	1 [Reference]	NA	1 [Reference]	NA	1 [Reference]	NA	1 [Reference]	NA
151-200	1.02 (0.99-1.05)	.16	1.01 (0.99-1.04)	.403	0.84 (0.81-0.86)	<.001	0.81 (0.78-0.84)	<.001
201-250	1.18 (1.15-1.21)	<.001	1.17 (1.13-1.20)	<.001	0.84 (0.81-0.87)	<.001	0.80 (0.77-0.83)	<.001
>250	1.59 (1.54-1.65)	<.001	1.57 (1.52-1.62)	<.001	1.11 (1.06-1.17)	<.001	1.05 (1.00-1.10)	.06
HDL-C, per 10-mg/dL increase	0.93 (0.92-0.94)	<.001	0.93 (0.92-0.94)	<.001	1.03 (1.02-1.04)	<.001	1.03 (1.02-1.04)	<.001
Systolic blood pressure, per 10–mm Hg increase	1.09 (1.09-1.10)	<.001	1.09 (1.09-1.10)	<.001	1.06 (1.06-1.07)	<.001	1.06 (1.06-1.07)	<.001
Blood pressure treatment vs no treatment	1.35 (1.33-1.39)	<.001	1.37 (1.35-1.39)	<.001	1.39 (1.35-1.41)	<.001	1.45 (1.41-1.49)	<.001
Statin treatment vs no treatment	NA	NA	0.93 (0.91-0.95)	<.001	NA	NA	0.75 (0.72-0.77)	<.001

Abbreviations: ASCVD, atherosclerotic cardiovascular disease; HDL-C, high-density lipoprotein cholesterol; HR, hazard radio; NA, not applicable.

SI conversion factor: To convert total cholesterol and HDL-C to millimoles per liter, multiply by 0.0259.

Table 2 also shows the results of 5-year prediction models for ASCVD death. Again, risk factors included increasing age (without statin: HR, 1.51; 95% CI, 1.50-1.52; *P* < .001; with statin: HR, 1.51; 95% CI, 1.50-1.52; *P* < .001), male sex (women without statin: HR, 0.58; 95% CI, 0.54-0.63; *P* < .001; with statin: HR, 0.58; 95% CI, 0.54-0.63; *P* < .001), black race (without statin: HR, 1.20; 95% CI, 1.16-1.24; *P* < .001; with statin: HR, 1.17; 95% CI, 1.13-1.21; *P* < .001), diabetes (without statin: HR, 1.32; 95% CI, 1.28-1.35; *P* < .001; with statin: HR, 1.36; 95% CI, 1.33-1.40; *P* < .001), current smoking (without statin: HR, 1.24; 95% CI, 1.19-1.28; *P* < .001; with statin: HR, 1.22; 95% CI, 1.18-1.26; *P* < .001), and antihypertensive therapy (without statin: HR, 1.39; 95% CI, 1.35-1.41; *P* < .001; with statin: HR, 1.45; 95% CI, 1.41-1.49; *P* < .001). Statin therapy at baseline was associated with a 25% (95% CI, 23%-28%) lower relative risk for ASCVD death (*P* < .001).

The directions and magnitudes of risk factor effects were similar in stratified 5-year analyses of patients younger than 65 years (eTable 3 in the Supplement) and those aged 65 years or older (eTable 4 in the Supplement). Statin therapy was associated with lower relative risk of ASCVD death in both age strata but was only associated with lower relative risk of composite ASCVD events among the older stratum (<65 years: HR, 1.00; 95% CI, 0.97-1.00; *P* = .89; ≥65 years: HR, 0.87; 95% CI, 0.84-0.89; *P* < .001).

The HRs for the risk factors were similar in the 10-year models for ASCVD events and mortality (eTable 5 in the Supplement) compared with the 5-year models (Table 2). A notable exception was smoking status, which was associated with a higher risk of ASCVD events (HR, 1.68; 95% CI, 1.65-1.70; *P* < .001) and mortality (HR, 1.74; 95% CI, 1.70-1.77; *P* < .001) during a 10-year period than during a 5-year period (HR, 1.52; 95% CI, 1.49-1.55; *P* < .001 and HR, 1.24; 95% CI, 1.19-1.28; *P* < .001, respectively).

As shown in Table 3, the use of cohort-specific effect estimates (model 2) and the addition of statin therapy (model 3) did not meaningfully improve discrimination for risk of ASCVD event in any of the 4 sex-race groups examined compared to the PCE model (model 1) (eg, Harrell C statistic [SD] among white men, model 1: 0.66 [0.004]; model 2, 0.67 [0.004]; model 3: 0.67 [0.004]). All models had better discrimination among women than among men (eg, Harrell C statistic [SD] among black women vs black men, model 1: 0.79 [0.036] vs 0.72 [0.007]; model 2: 0.80 [0.030] vs 0.76 [0.007]; model 3: 0.80 [0.029] vs 0.72 [0.007]). These patterns were observed in the overall cohort and the subset aged 40 to 79 years (Table 3).

**Table 3.** Harrell C Statistics for Composite ASCVD Events

Model	C statistic (SD)			
	Men		Women	
	White	Black	White	Black
**Overall cohort of 1 672 336 veterans**				
ASCVD events, No.	54 550	10 575	1154	326
No. at risk	1 314 938	260 225	69 055	28 118
Model 1, 2013 PCE	0.66 (0.004)	0.72 (0.007)	0.78 (0.020)	0.79 (0.036)
Model 2, 2013 PCE with cohort-derived β	0.67 (0.004)	0.72 (0.007)	0.80 (0.018)	0.80 (0.030)
Model 3, 2013 PCE with cohort-derived β and statin therapy	0.67 (0.004)	0.72 (0.007)	0.80 (0.018)	0.80 (0.029)
**Subset aged 40-79 y with 1 415 057 veterans**				
ASCVD events, No.	48 169	9609	847	285
No. at risk	1 136 161	218 463	44 399	16 034
Model 1, 2013 PCE	0.63 (0.004)	0.68 (0.008)	0.72 (0.022)	0.72 (0.045)
Model 2, 2013 PCE with cohort-derived β	0.64 (0.004)	0.68 (0.008)	0.73 (0.023)	0.73 (0.038)
Model 3, 2013 PCE with cohort-derived β and statin therapy	0.64 (0.004)	0.68 (0.008)	0.73 (0.023)	0.73 (0.036)

Abbreviations: ASCVD, atherosclerotic cardiovascular disease; PCE, Pooled Cohort Equation.

**Figure 2.**
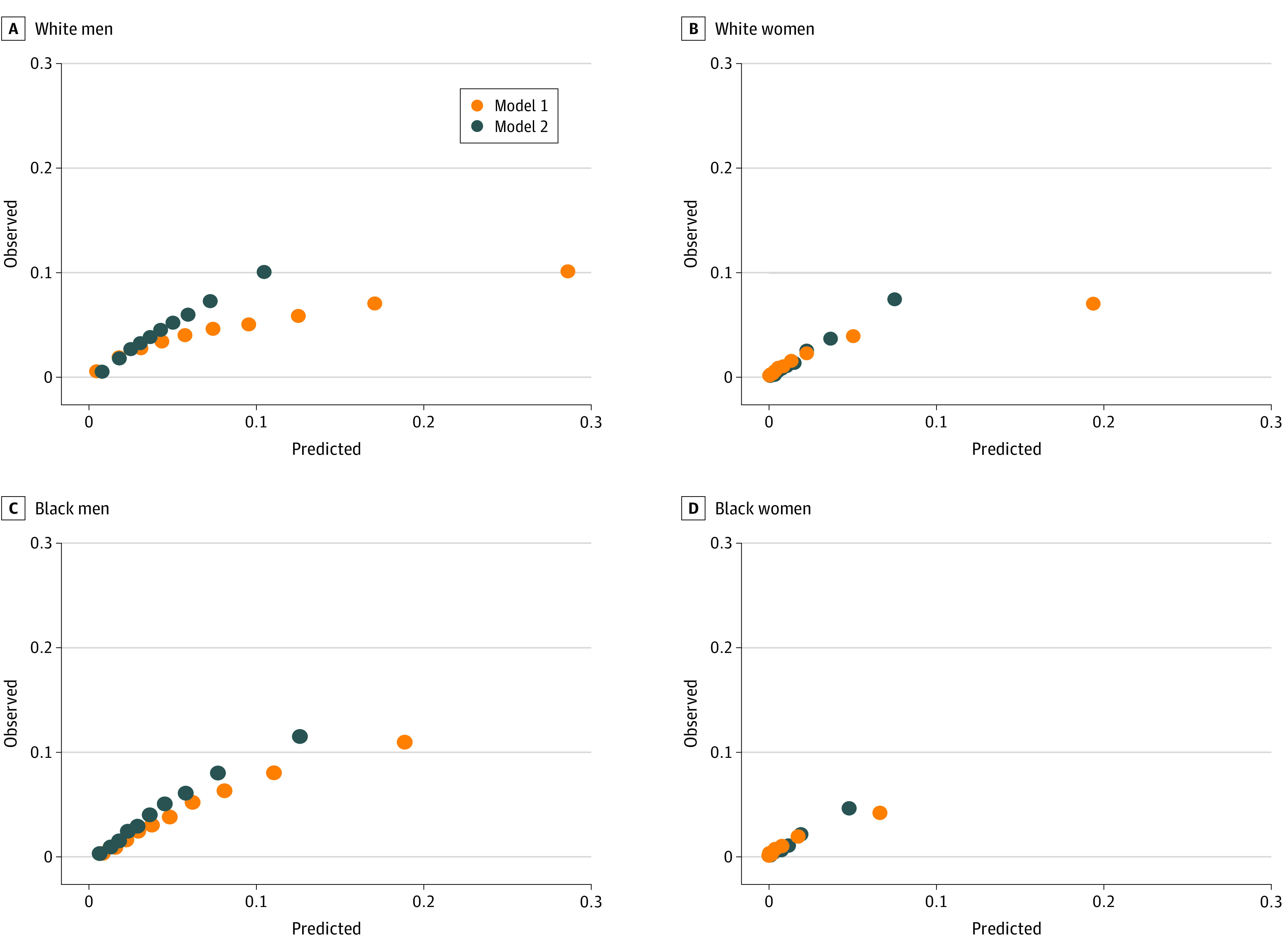
Calibration of Pooled Cohort Equations and Cohort-Derived Model for 5-Year Prediction of Atherosclerotic Cardiovascular Disease Events

Figure 2 illustrates the calibration of models 1 and 2 for 5-year ASCVD estimation among the 4 race-sex strata. In all strata, the PCE model (model 1) overestimated 5-year event rates, with all slopes of observed vs expected event rates deviating considerably from 1. The use of cohort-derived effect estimates (model 2) improved calibration. eTable 6 in the Supplement shows the distribution of patients aged 40 to 79 years in clinically relevant categories of 5-year ASCVD risk by the PCE (model 1) and the cohort-specific model (model 2). In the cohort overall, 211 237 of 1 136 161 white men (18.6%) and 29 634 of 218 463 black men (13.6%) would have an estimated 5-year ASCVD risk of at least 3.75% according to the PCE and thus be potentially eligible for statin therapy when the cohort-specific model categorized their 5-year risk as less than 3.75%. Similarly, 1741 of 44 399 white women (3.9%) and 836 of 16 034 black women (5.2%) were assigned higher risk categories by the PCE compared with the cohort-specific model. The risk calculator for the VA cohort-specific model is publicly avaialble.^17^

## Discussion

We have shown that the 2013 PCE overestimated absolute ASCVD risk in a contemporary patient cohort receiving real-world care in a large national health care system and that the use of cohort-specific estimates for PCE risk factors improved model calibration and assignment to clinically meaningful risk categories. Baseline statin therapy was associated with an 8% lower 5-year ASCVD relative risk and 26% lower 5-year relative risk of ASCVD mortality, but the addition of statin therapy to models did not improve discrimination. These results call into question the validity of applying ASCVD prediction models developed in the prestatin era to contemporary ASCVD risk estimation, but they do not necessitate the addition of statin therapy to these models.

Risk stratification is a critical first step for implementing primary ASCVD prevention, and key interventions include lifestyle modification and pharmacotherapy with statins and aspirin. Statins have demonstrated conclusive benefits for reducing ASCVD events and mortality in randomized clinical trials.^18,19^ The 2013 Cardiovascular Risk Estimation Guidelines estimated that 10-year ASCVD risk exceeded 7.5% for approximately one-third of US adults and designated such individuals as definite candidates for statin therapy. As a result, more US residents now receive statin therapy.^5^ Given that the PCE were developed in cohorts from the late 1960s through the early 1990s,^20^ it is reasonable to evaluate whether the PCE and other similarly dated models remain clinically valid for contemporary risk estimation among patient populations heavily enriched with statin users. The present analyses demonstrated overestimation of ASCVD risk based on PCE. Such an approach in the modern era may lead to inappropriate ASCVD risk estimation and might affect how preventive therapy is instituted.

The inadequacy of the PCE for ASCVD risk estimation in the present cohort is a matter of poor model calibration, a measure of how closely the absolute event rates estimated by a model match the rates actually observed.^21^ Since current primary prevention guidelines use categories of absolute ASCVD risk (eg, 7.5%-19.9% 10-year risk) to guide recommendations for statin therapy,^22^ calibration is an important metric to inform the ASCVD prevention programs in a health care system. We found that the published PCE would significantly overestimate ASCVD risk in the national VA patient population. Approximately 1 in 6 men in this cohort had PCE-estimated ASCVD risk that would make them potentially eligible for statin treatment and cohort-estimated risk that would not. Given this overestimation, we tested the hypothesis and demonstrated that deriving new cohort-specific effect estimates for the original PCE risk factors improved model calibration, correcting this overestimation.

The present findings from a health care system–derived cohort are consistent with a meta-analysis of data from 360 737 participants in 86 prospective studies in the Emerging Risk Factors Collaboration,^23^ in which the PCE overestimated ASCVD relative risk by a mean of 41%. Indeed, the PCE overestimated ASCVD in the cohorts used for the original PCE model validation.^2,20^ Our national results are also consistent with those from more limited health care patient populations from Florida, Illinois, and Minnesota^24,25^ and from a prior report of national VA data, which found that the PCE estimated 63% more ASCVD events than were observed.^7^ The present study extends this latter work by using a novel high-throughput phenotyping algorithm to identify ASCVD events, deriving new effect estimates for PCE terms, and evaluating the inclusion of statin therapy in risk estimation. Our demonstration that recalculating cohort-derived β coefficients for PCE terms improves PCE model performance in a specific population is consistent with an analysis of electronic health record data from more than 84 000 adults receiving care at a large health care delivery and insurance organization in Minnesota between 2001 and 2011.^25^ Another approach to correcting the risk overestimation by the PCE is model recalibration, whereby observed risk in a target population is used to recalculate the model’s overall slope, intercept, or both.^20,26,27^ Recalibration of the PCE successfully corrected the observed risk overestimation in the Emerging Risk Factors Collaboration and the prior VA study.^7,23^ However, recalibration may only be appropriate when risk factors have consistent directions of effect between cohorts. The nonmonotonic association we observed between total cholesterol and ASCVD outcomes in this VA cohort favors our approach of reestimating the effects.

Beyond the derivation of new cohort-specific effect estimates for risk factors in the PCE, it is reasonable to ask whether statin therapy itself should be included in ASCVD models in the modern era. In this contemporary national cohort receiving real-world clinical care, baseline statin therapy in veterans was associated with a clinically significant reduction in 5-year ASCVD events and mortality, consistent with evidence from randomized clinical trials.^18,28^ We limited our analyses to a 5-year period instead of the 10 years used by the PCE to reduce the potential consequences of incident (ie, drop-in) statin use after baseline, which occurred for 54% of participants within 5 years and for 63% of participants within 10 years. Nevertheless, we observed lower-than-anticipated ASCVD event rates in a contemporary cohort enriched for ASCVD risk factors. Prevalent and drop-in statin use during follow-up in this cohort is likely to be a major determinant of these lower rates and suggests the need for policy makers and clinicians to consider shortening the time horizon of the models used to inform ASCVD prevention. Given that more than half of the participants were prescribed statin therapy within 5 years of baseline, whether a 5-year or even shorter period is the optimal interval remains an open question.

This research has clinical implications for ASCVD prevention in modern medicine. Before the widespread implementation of electronic health records and clinical databases, clinicians had to rely on ASCVD risk models from external cohort studies to estimate the risk for the patients under their care. It is now becoming increasingly feasible for clinicians practicing in large health care systems to use patient data from their specific settings to estimate ASCVD risk more accurately.^25^ The present work demonstrates the potential advantage of using risk estimation models derived from one’s own practice setting to guide ASCVD prevention, even in a large diverse health care system spanning all regions of the United States. Our investigation offers insight into how the VA health care system and other large integrated systems might approach precision ASCVD prevention for their patient populations.

### Limitations

Our work has some limitations to note. First, only 6% of the cohort were women, although this proportion represents almost 100 000 individuals and 1500 ASCVD events. Our models demonstrated better discrimination among women than men, although the 95% CIs around these estimates indicated lower precision. Second, our analysis did not incorporate certain key patient characteristics that define statin eligibility by current guidelines, such as risk-enhancing factors like coronary artery calcium score or shared decision-making.^4^ Third, we limited our analysis to an examination of the PCE and its risk factors and did not undertake the development and validation of a novel risk prediction model.

## Conclusions

In this study, we observed lower rates of incident ASCVD events than expected in a contemporary national cohort. The PCE overestimated ASCVD risk, and more than 20% of patients would potentially be eligible for statin therapy based on the PCE but not on a cohort-derived model. In the statin era, health care professionals and systems should base ASCVD risk assessment on models calibrated to their patient populations.

## Notes

Supplement. **eFigure. **Study Flow Diagram **eTable 1. **Cohort Exclusion Criteria and Definitions of Diabetes and Statin Use **eTable 2. **Baseline Characteristics and Incident ASCVD Events Among 1 663 422 Veterans for 10-Year Risk Prediction **eTable 3. **5-Year Prediction Models for Composite ASCVD Events and ASCVD Mortality, Without and With Statin Therapy, Age <65 **eTable 4. **5-Year Prediction Models for Composite ASCVD Events and ASCVD Mortality, Without and With Statin Therapy, Age ≥65 **eTable 5. **10-Year Prediction Models for Composite ASCVD Events and ASCVD Mortality, Without and With Statin Therapy **eTable 6. **Distribution of Patients Aged 40-79 Years Into Clinically Relevant 5-Year ASCVD Risk Categories by the PCE and Cohort-Derived Models
