# Supplementary material for: Estimation of Atherosclerotic Cardiovascular Disease Risk Among Patients in the Veterans Affairs Health Care System
Source: JAMA Netw Open. 2020 Jul 14;3(7):e208236. doi: 10.1001/jamanetworkopen.2020.8236 (PMC7361654; doi:10.1001/jamanetworkopen.2020.8236)
Supplement: Supplement. — eFigure. Study Flow Diagram eTable 1. Cohort Exclusion Criteria and Definitions of Diabetes and Statin Use eTable 2. Baseline Characteristics and Incident ASCVD Events Among 1 663 422 Veterans for 10-Year Risk Prediction eTable 3. 5-Year Prediction Models for Composite ASCVD Events and ASCVD Mortality, Without and With Statin Therapy, Age <65 eTable 4. 5-Year Prediction Models for Composite ASCVD Events and ASCVD Mortality, Without and With Statin Therapy, Age ≥65 eTable 5. 10-Year Prediction Models for Composite ASCVD Events and ASCVD Mortality, Without and With Statin Therapy eTable 6. Distribution of Patients Aged 40-79 Years Into Clinically Relevant 5-Year ASCVD Risk Categories by the PCE and Cohort-Derived Models [file jamanetwopen-3-e208236-s001.pdf]

Supplementary Online Content

Vassy JL, Lu B, Ho Y-L, et al. Estimation of atherosclerotic cardiovascular disease risk among patients in the Veterans Affairs health care system. *JAMA Netw Open*. 2020;3(7):e208236. doi:10.1001/jamanetworkopen.2020.8236

eFigure. Study Flow Diagram

eTable 1. Cohort Exclusion Criteria and Definitions of Diabetes and Statin Use

eTable 2. Baseline Characteristics and Incident ASCVD Events Among 1 663 422 Veterans for 10-Year Risk Prediction

eTable 3. 5-Year Prediction Models for Composite ASCVD Events and ASCVD Mortality, Without and With Statin Therapy, Age <65

eTable 4. 5-Year Prediction Models for Composite ASCVD Events and ASCVD Mortality, Without and With Statin Therapy, Age ≥65

eTable 5. 10-Year Prediction Models for Composite ASCVD Events and ASCVD Mortality, Without and With Statin Therapy

eTable 6. Distribution of Patients Aged 40-79 Years Into Clinically Relevant 5-Year ASCVD Risk Categories by the PCE and Cohort-Derived Models

This supplementary material has been provided by the authors to give readers additional information about their work.

© 2020 Vassy JL et al. *JAMA Network Open.*

eFigure. Study Flow Diagram

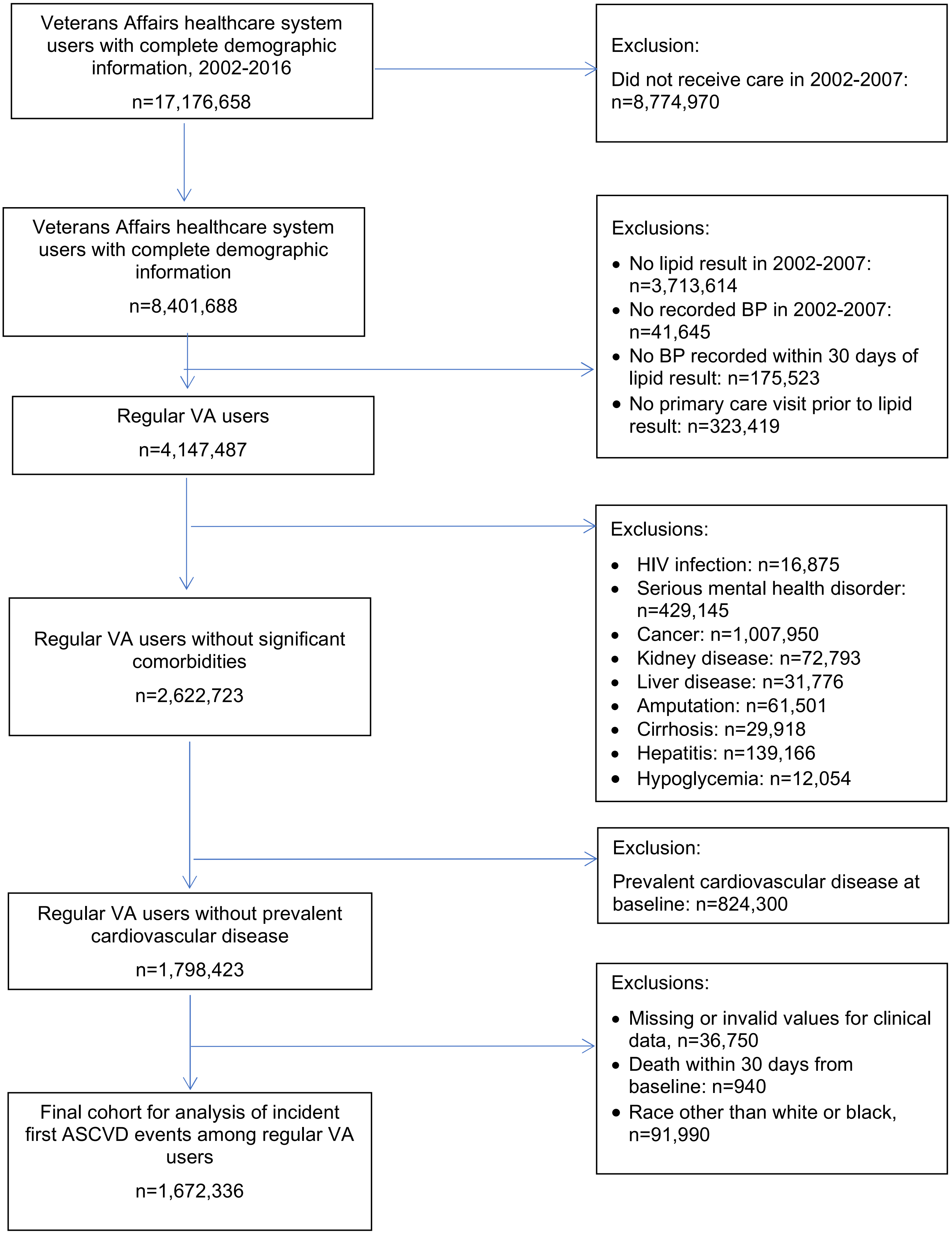

© 2020 Vassy JL et al. *JAMA Network Open.*

**eTable 1.** Cohort Exclusion Criteria and Definitions of Diabetes and Statin Use		
**Rule**	**Concept**	**Values**
**Exclusion criteria A: 1 inpatient OR 2 outpatient codes**	HIV positivity	ICD-9: 042, 795.71
	Chronic kidney disease or end-stage renal disease	ICD-9: 403.01, 403.11, 403.91, 404.02, 404.03, 404.12, 404.13, 404.92, 404.93; 585.5, 585.6, V45.1, V56.xx, 996.73<br>Procedure code 38.95
	Liver disease or hepatitis	ICD-9: 571.0, 571.1, 571.2, 571.3, 571.4, 571.5, 571.6, 571.8, 571.9, 573.3, 070.1, 070.2, 070.3, 070.4, 070.5, 070.6, 070.7, 070.8, 070.9
	Cancer, other than non-melanoma skin cancer	ICD-9: 140.00-239.00, except 173.2, 173.4 and 173.9
	Schizophrenia	ICD-9: 295, 301.2, 313.2
	Dementia	ICD-9: 290.x, 291.2, 294.1, 331.x (excluding 331.83), 333.0, 333.4, 797.x, 332.0, 294.8, 046.1, 046.3
**Exclusion criterion B: 1 inpatient or outpatient code**	Amputation	ICD-9: V49.7x<br>CPT: 24900, 24920, 24930, 27025, 27027, 27122, 27215-27218, 27226-27228, 27235, 27236, 27244, 27245, 27248, 27254, 27590, 27594, 27880, 27881, 27886, 27888, 28805<br>VA-rated disability condition: ‘Amputation’, ‘Prosthetic’
**Diabetes: medication prior to baseline AND either 2 ICD code OR 1 ICD code at a primary care visit**	Diabetes medication	Exenatide, insulin, acetohexamide, alogliptin, canagliflozin, chlorpropamide, dapagliflozin, empagliflozin, glimepiride, glipizide, glyburide, linagliptin, metformin, nateglinide, pioglitazone, repaglinide, rosiglitazone, saxagliptin, sitagliptin, tolazamide, tolbutamide, troglitazone, albiglutide, dulaglutide, liraglutide, lixisenatide, pramlintide
	Diabetes diagnosis	ICD-9 250.XX
**Statin use**	Statin prescription	Atorvastatin, cerivastatin, fluvastatin, lovastatin, pitavastatin, pravastatin, rosuvastatin, simvastatin

Abbreviations: CPT, Current Procedural Terminology; ICD, International Classification of Disease

© 2020 Vassy JL et al. *JAMA Network Open.*

eTable 2. Baseline Characteristics and Incident ASCVD Events Among 1 663 422 Veterans for 10-Year Risk Prediction

Characteristics	Total cohort (n=1,663,422)	ASCVD event (n=121,893)	No ASCVD event (n=1,541,529)	ASCVD death (n=74,230)	No ASCVD death (n=1,589,192)
Age, mean (SD), y	58.0 (13.7)	63.4 (11.6)	57.6 (13.8)	69.5 (11.7)	57.5 (13.6)
Total cholesterol, mean (SD), mg/dL	197.1 (40.6)	199.1 (42.2)	197.0 (39.9)	193.0 (41.1)	197.3 (40.0)
HDL-C, mean (SD), mg/dL	45.9 (14.0)	44.6 (14.2)	46.0 (14.0)	46.1 (15.0)	45.9 (14.0)
Systolic blood pressure, mean (SD), mm Hg	135.7 (18.3)	141.2 (20.2)	135.3 (18.1)	142.3 (20.1)	135.4 (18.1)
Male, No. (%)	1,566,616 (94.2)	118,922 (97.6)	1,1447,694 (93.9)	72,608 (97.8)	1,494,008 (94.0)
White, No. (%)	1,376,108 (82.7)	101,028 (82.9)	1,275,080 (82.7)	64,205 (86.5)	1,311,903 (82.6)
Current smoker, No. (%)	331,384 (19.9)	28,080 (23.0)	303,304 (19.7)	12,495 (16.8)	318,889 (20.1)
Diabetes, No. (%)	288,807 (17.4)	35,153 (28.8)	253,654 (16.5)	20,019 (27.0)	268,788 (16.9)
Blood pressure treatment, No. (%)	680,160 (40.9)	68,567 (56.3)	611,593 (39.7)	45,279 (61.0)	634,881 (39.9)
Statin treatment, No. (%)	310,863 (18.7)	27,589 (22.6)	283,274 (18.4)	16,522 (22.3)	294,341 (18.5)

Abbreviations: HDL-C, high-density lipoprotein cholesterol.
SI conversion factors: To convert total cholesterol and HDL-C to mmol/L, multiply values by 0.0259. To convert systolic blood pressure to kPa, multiply values by 0.133

© 2020 Vassy JL et al. *JAMA Network Open.*

**eTable 3.** 5-Year Prediction Models for Composite ASCVD Events and ASCVD Mortality, Without and With Statin Therapy, Age <65								
	**Composite ASCVD events**				**ASCVD mortality**			
	**Without statin**		**With statin**		**Without statin**		**With statin**	
**Characteristics**	**HR (95% CI)**	**p**	**HR (95% CI)**	***p***	**HR (95% CI)**	**p**	**HR (95% CI)**	***p***
Age, per 5 years	1.29 (1.28, 1.30)	<0.001	1.30 (1.29, 1.31)	<0.001	1.33 (1.31, 1.35)	<0.001	1.34(1.32, 1.36)	<0.001
Female, vs. male	0.60 (0.57, 0.65)	<0.001	0.60 (0.57, 0.65)	<0.001	0.36 (0.30, 0.41)	<0.001	0.36 (0.30, 0.41)	<0.001
Black race, vs. white	1.11 (1.08, 1.14)	<0.001	1.11 (1.08, 1.14)	<0.001	1.03 (0.98, 1.08)	0.25	1.01 (0.96, 1.06)	0.42
Diabetes, vs. no diabetes	1.72 (1.68, 1.77)	<0.001	1.72 (1.68, 1.77)	<0.001	1.47 (1.40, 1.54)	<0.001	1.52 (1.45, 1.59)	<0.001
Currently smoking vs. not currently smoking	1.58 (1,54, 1.61)	<0.001	1.58 (1.54, 1.61)	<0.001	1.23 (1.17, 1.28)	<0.001	1.22 (1.16, 1,27)	<0.001
Total cholesterol strata, mg/dL								
50-150	1.00		1.00		1.00		1.00	
151-200	1.03 (0.99, 1.07)	0.13	1.03 (0.99, 1.07)	0.13	0.81 (0.76, 0.86)	<0.001	0.79 (0.74, 0.84)	<0.001
201-250	1.19 (1.15, 1.24)	<0.001	1.19 (1.14, 1.24)	<0.001	0.80 (0.74, 0.85)	<0.001	0.77 (0.72, 0.82)	<0.001
>250	1.63 (1.56, 1.70)	<0.001	1.63 (1.56, 1.70)	<0.001	1.05 (0.97, 1.13)	0.27	1.01 (0.94, 1.10)	0.77
HDL-C, per 10 mg/dL	0.93 (0.92, 0.94)	<0.001	0.93 (0.92, 0.94)	<0.001	1.08 (1.07, 1.10)	<0.001	1.08 (1.07, 1.10)	<0.001
Systolic blood pressure, per 10 mmHg	1.12 (1.12, 1.13)	<0.001	1.12 (1.12, 1.13)	<0.001	1.13 (1.12, 1.15)	<0.001	1.13 (1.12, 1.14)	<0.001
Blood pressure treatment, vs. no treatment	1.43 (1.39,1.45)	<0.001	1.43 (1.39, 1.45)	<0.001	1.61 (1.56, 1.69)	<0.001	1.69 (1.61, 1.75)	<0.001
Statin treatment, vs. no treatment	-	-	1.00 (0.97, 1.00)	0.89	-	-	0.76 (0.72, 0.80)	<0.001

Abbreviations: CI, confidence interval; HDL-C, high-density lipoprotein cholesterol; HR, hazard radio.
SI conversion factors: To convert total cholesterol and HDL-C to mmol/L, multiply values by 0.0259. To convert systolic blood pressure to kPa, multiply values by 0.133.

© 2020 Vassy JL et al. *JAMA Network Open.*

**eTable 4.** 5-Year Prediction Models for Composite ASCVD Events and ASCVD Mortality, Without and With Statin Therapy, Age ≥65								
	**Composite ASCVD events**				**ASCVD mortality**			
	**Without statin**		**With statin**		**Without statin**		**With statin**	
**Characteristics**	**HR (95% CI)**	**p**	**HR (95% CI)**	***p***	**HR (95% CI)**	**p**	**HR (95% CI)**	***p***
Age, per 5 years	1.33 (1.31, 1.34)	<0.001	1.32 (1.31, 1.33)	<0.001	1.75 (1.73, 1.77)	<0.001	1.73 (1.71, 1.75)	<0.001
Female, vs. male	0.76 (0.70, 0.82)	<0.001	0.76 (0.70, 0.83)	<0.001	0.66 (0.60, 0.72)	<0.001	0.66 (0.60, 0.73)	<0.001
Black race, vs. white	1.41 (1.36, 1.46)	<0.001	1.39 (1.34, 1.44)	<0.001	1.23(1.17, 1.28)	<0.001	1.20 (1.15, 1.26)	<0.001
Diabetes, vs. no diabetes	1.46 (1.43, 1.50)	<0.001	1.48 (1.44, 1.52)	<0.001	1.30 (1.26, 1.34)	<0.001	1.33 (1.29, 1.37)	<0.001
Currently smoking vs. not currently smoking	1.27 (1.21, 1.32)	<0.001	1.26(1.21, 1.31)	<0.001	1.14 (1.08, 1.21)	<0.001	1.13(1.07, 1.19)	0.73
Total cholesterol strata, mg/dL								
50-150	1.00		1.00		1.00		1.00	
151-200	1.00 (0.97, 1.04)	0.97	0.98 (0.95, 1.02)	0.340	0.87 (0.83, 0.90)	<0.001	0.84 (0.81, 0.87)	<0.001
201-250	1.14 (1.10, 1.18)	<0.001	1.10 (1.06, 1.15)	<0.001	0.89 (0.85, 0.93)	<0.001	0.84 (0.80, 0.88)	<0.001
>250	1.40 (1.33, 1.48)	<0.001	1.35 (1.28, 1.43)	<0.001	1.13 (1.06, 1.21)	<0.001	1.07 (1.00, 1.14)	0.06
HDL-C, per 10 mg/dL	0.93 (0.92, 0.94)	<0.001	0.93 (0.93, 0.94)	<0.001	1.00 (0.99, 1.01)	0.39	1.00 (0.99, 1.01)	0.94
Systolic blood pressure, per 10 mmHg	1.06 (1.05, 1.07)	<0.001	1.06 (1.05, 1.07)	<0.001	1.03 (1.03, 1.04)	<0.001	1.03 (1.03, 1.04)	<0.001
Blood pressure treatment, vs. no treatment	1.25 (1.22, 1.28)	<0.001	1.28 1.25,1.32)	<0.001	1.30 (1.27, 1.33)	<0.001	1.35 (1.30, 1.39)	<0.001
Statin treatment, vs. no treatment	-	-	0.87 (0.84, 0.89)	<0.001	-	-	0.78 (0.76, 0.81)	<0.001

Abbreviations: CI, confidence interval; HDL-C, high-density lipoprotein cholesterol; HR, hazard radio.
SI conversion factors: To convert total cholesterol and HDL-C to mmol/L, multiply values by 0.0259. To convert systolic blood pressure to kPa, multiply values by 0.133.

© 2020 Vassy JL et al. *JAMA Network Open.*

eTable 5. 10-Year Prediction Models for Composite ASCVD Events and ASCVD Mortality, Without and With Statin Therapy

	**Composite ASCVD events**				**ASCVD mortality**			
	**Without statin**		**With statin**		**Without statin**		**With statin**	
**Characteristics**	**HR (95% CI)**	**p**	**HR (95% CI)**	***p***	**HR (95% CI)**	**p**	**HR (95% CI)**	***p***
Age, per 5 years	1.22 (1.22, 1.23)	<0.001	1.22 (1.22, 1.23)	<0.001	1.54 (1.53, 1.55)	<0.001	1.54 (1.53, 1.54)	<0.001
Female, vs. male	0.66 (0.64, 0.69)	<0.001	0.66 (0.64, 0.69)	<0.001	0.67 (0.63, 0.70)	<0.001	0.67 (0.63, 0.70)	<0.001
Black race, vs. white	1.21 (1.20, 1.23)	<0.001	1.21 (1.19, 1.23)	<0.001	1.18 (1.15, 1.20)	<0.001	1.15 (1.13, 1.18)	<0.001
Diabetes, vs. no diabetes	1.63 (1.61, 1.65)	<0.001	1.65 (1.62, 1.67)	<0.001	1.41 (1.39, 1.44)	<0.001	1.45 (1.42, 1.47)	<0.001
Current smoking, vs. not currently smoking	1.68 (1.65, 1.70)	<0.001	1.67(1.65, 1.70)	<0.001	1.74 (1.70, 1.77)	<0.001	1.72 (1.69, 1.76)	<0.001
Total cholesterol strata, mg/dL								
50-150	Ref		Ref		Ref		Ref	
151-200	1.03 (1.02, 1.05)	<0.05	1.03 (1.01, 1.05)	<0.05	0.87 (0.85, 0.89)	<0.001	0.85 (0.83, 0.86)	<0.001
201-250	1.19 (1.16, 1.21)	<0.001	1.17 (1.15, 1.19)	<0.001	0.88 (0.86, 0.90)	<0.001	0.84 (0.82, 0.86)	<0.001
>250	1.53 (1.50, 1.57)	<0.001	1.51 (1.47, 1.55)	<0.001	1.10 (1.06, 1.13)	<0.001	1.04 (1.01, 1.08)	<0.05
HDL-C, per 10 mg/dL	0.94 (0.93, 0.94)	<0.001	0.94 (0.93, 0.94)	<0.001	1.01 (1.01, 1.02)	<0.05	1.01 (1.01, 1.02)	<0.001
Systolic blood pressure, per 10 mmHg	1.09 (1.09, 1.10)	<0.001	1.09 (1.09, 1.10)	<0.001	1.07 (1.07, 1.08)	<0.001	1.07 (1.07, 1.08)	<0.001
Blood pressure treatment, vs. no treatment	1.33 (1.32, 1.35)	<0.001	1.35 (1.33,1.37)	<0.001	1.37 (1.35, 1.39)	<0.001	1.43 (1.39,1.45)	<0.001
Statin treatment, vs. no treatment	-	-	0.92 (0.91, 0.94)	<0.001	-	-	0.79 (0.78, 0.80)	<0.001

Abbreviations: CI, confidence interval; HDL-C, high-density lipoprotein cholesterol; HR, hazard radio.
SI conversion factors: To convert total cholesterol and HDL-C to mmol/L, multiply values by 0.0259. To convert systolic blood pressure to kPa, multiply values by 0.133.

© 2020 Vassy JL et al. *JAMA Network Open.*

eTable 6. Distribution of Patients Aged 40-79 Years Into Clinically Relevant 5-Year ASCVD Risk Categories by the PCE and Cohort-Derived Models

	5-year ASCVD RISK	VA cohort-derived model No. (%)						VA cohort-derived model No. (%)				
**WHITE MEN**		**0-2.5%**	**2.5%-3.75%**	**3.75%-10%**	**>10%**	**TOTAL**	**BLACK MEN**	**0-2.5%**	**2.5%-3.75%**	**3.75%-10%**	**>10%**	**TOTAL**
**PCE No. (%)**	**0-2.5%**	156958<br>13.81	4575<br>0.40	0<br>0.00	0<br>0.00	**161533<br>14.22**	**0-2.5%**	43062<br>19.71	136<br>0.06	0<br>0.00	0<br>0.00	**43198<br>19.77**
	**2.5%-3.75%**	61987<br>5.46	67035<br>5.90	106<br>0.01	0<br>0.00	**129128<br>11.37**	**2.5%-3.75%**	20314<br>9.30	18905<br>8.65	241<br>0.11	0<br>0.00	**39460<br>18.06**
	**3.75%-10%**	1663<br>0.15	209488<br>18.44	268463<br>23.63	0<br>0.00	**479614<br>42.21**	**3.75%-10%**	1290<br>0.59	28344<br>12.97	64847<br>29.68	35<br>0.02	**94516<br>43.26**
	**>10%**	0<br>0.00	86<br>0.01	323337<br>28.46	42463<br>3.74	**365886<br>32.20**	**>10%**	0<br>0.00	0<br>0.00	25435<br>11.64	15854<br>7.26	**41289<br>18.90**
	**TOTAL**	**220608<br>19.42**	**281184<br>24.75**	**591906<br>52.10**	**42463<br>3.74**	**1136161<br>100.00**	**TOTAL**	**64666<br>29.60**	**47385<br>21.69**	**90523<br>41.44**	**15889<br>7.27**	**218463<br>100.00**
**WHITE WOMEN**							**BLACK WOMEN**					
**PCE No. (%)**	**0-2.5%**	31912<br>71.88	1662<br>3.74	7<br>0.02	0<br>0.00	**33581<br>75.63**	**0-2.5%**	11792<br>73.54	279<br>1.74	16<br>0.10	0<br>0.00	**12087<br>75.38**
	**2.5%-3.75%**	724<br>1.63	2170<br>4.89	670<br>1.51	0<br>0.00	**3564<br>8.03**	**2.5%-3.75%**	675<br>4.21	648<br>4.04	83<br>0.52	0<br>0.00	**1406<br>8.77**
	**3.75%-10%**	198<br>0.45	1422<br>3.20	3391<br>7.64	14<br>0.03	**5025<br>11.32**	**3.75%-10%**	117<br>0.73	702<br>4.38	1182<br>7.37	1<br>0.01	**2002<br>12.49**
	**>10%**	4<br>0.01	117<br>0.26	1731<br>3.90	377<br>0.85	**2229<br>5.02**	**>10%**	4<br>0.02	13<br>0.08	368<br>2.30	154<br>0.96	**539<br>3.36**
	**TOTAL**	**32838<br>73.96**	**5371<br>12.10**	**5799<br>13.06**	**391<br>0.88**	**44399<br>100.00**	**TOTAL**	**12588<br>78.51**	**1642<br>10.24**	**1649<br>10.28**	**155<br>0.97**	**16034<br>100.00**

Abbreviations: ASCVD, atherosclerotic cardiovascular disease; PCE, pooled cohort equation.

© 2020 Vassy JL et al. *JAMA Network Open.*
